# Alluvial substrate mapping by automated texture segmentation of recreational-grade side scan sonar imagery

**DOI:** 10.1371/journal.pone.0194373

**Published:** 2018-03-14

**Authors:** Daniel Hamill, Daniel Buscombe, Joseph M. Wheaton

**Affiliations:** 1 Department of Watershed Sciences, Utah State University, Logan, UT, United States of America; 2 School of Earth Sciences and Environmental Sustainability, Northern Arizona University, Flagstaff, AZ, United States of America; University of Waikato, NEW ZEALAND

## Abstract

Side scan sonar in low-cost ‘fishfinder’ systems has become popular in aquatic ecology and sedimentology for imaging submerged riverbed sediment at coverages and resolutions sufficient to relate bed texture to grain-size. Traditional methods to map bed texture (i.e. physical samples) are relatively high-cost and low spatial coverage compared to sonar, which can continuously image several kilometers of channel in a few hours. Towards a goal of automating the classification of bed habitat features, we investigate relationships between substrates and statistical descriptors of bed textures in side scan sonar echograms of alluvial deposits. We develop a method for automated segmentation of bed textures into between two to five grain-size classes. Second-order texture statistics are used in conjunction with a Gaussian Mixture Model to classify the heterogeneous bed into small homogeneous patches of sand, gravel, and boulders with an average accuracy of 80%, 49%, and 61%, respectively. Reach-averaged proportions of these sediment types were within 3% compared to similar maps derived from multibeam sonar.

## Introduction

The grain size of bed sediment is a fundamental attribute of rivers and streams [1], and an important independent variable in studies of river adjustment [2], river classification [3], sediment transport [4], hydraulic roughness [5], and aquatic habitat [6] and therefore is an essential component of habitat suitability models [7–10].

Riverbeds are often arranged in sediment patch structures or facies of like-sediment [11] providing a diverse range of spatially coherent yet mobile micro-topographies [12]. Some sediment patches remain stationary because of large-scale topographic or obstruction-driven hydraulics [13], whereas others migrate freely in response to variable water and sediment supply [14]. Field studies have demonstrated how spatial variations in grain size affect the longitudinal organization of benthic community structure [15]. To develop suitable management and conservation strategies it is necessary to identify what controls the spatial variability in benthic habitats. The hierarchical organization of aquatic ecosystems [16] and the variable nature of grain size create a complex situation where, if relationships between aquatic organisms and grain size exist, these are highly non-linear linkages that are established across multiple scales [17]. Direct (and linear) relationships between aquatic organisms and grain size are often difficult to establish because grain size alone does not uniquely describe complex physical habitat [18], or alternatively because relating animal behavior in a spatially continuous sense to sediment relies on spatially continuous substrate maps, which traditionally are difficult to construct [17].

In recent years, side scan sonar within commodity ‘fishfinder’ systems have become an increasingly popular low-cost sensor for qualitative mapping of riverbed sediment and benthic environments [19–22]. We term these relatively low quality sonar systems, ‘recreational-grade’ to distinguish them from relatively higher quality, relatively expensive, ‘survey-grade’ side scan sonar systems [22]. In contrast to survey-grade side scan sonar, recreational-grade systems are typically operated on personal water crafts with out high-quality positioning and boat attitude (heave, pitch, roll, etc.) information. Kaeser et al. [23] demonstrated that recreational-grade side scan sonar imagery, called echograms, collected using a recreational-grade system in a riverine environment had sufficient detail to map locations of large woody debris. Subsequent studies have established that the resolution and quality of the echogram is sufficient to visually identify sediment facies [19–21] over reaches up to hundreds of kilometers in length, and these sonar have enjoyed a proliferation of use among aquatic ecologists [19–21, 23–30].

Despite allowing rapid collection high-resolution echograms across large areas, from which it is possible to visually identify sediment groupings, methods to automatically post-process and interpret data collected with a recreational-grade system are currently limited [22]. Recreational-grade systems are designed for providing images of the bed from a vessel, and do not record data to a hydrographic standard, or in standard data formats. Kaeser et al. [26] created a semi-automated, open-source GIS routine to create a georeferenced echogram by ‘rubbersheeting’ overlapping screenshots from the topside unit within a geographical information system platform, for subsequent visual interpretation. This methods works fairly well, but is labor intensive, subjective, and not practically applied to large-volumes of data. In addition, it does not correct for geometric or radiometric distortions present within the data. Buscombe [22] developed an open source program to automate the production of geometrically and radiometrically corrected georectified echograms directly from the binary files recorded by recreational-grade systems. Automated approaches to extracting and processing the data also presents the opportunity to draw upon automated side scan imaging processing literature [31–33] and to develop more objective approaches for carrying out spatially distributed substrate classification [34–37]. For highly heterogeneous sedimentary deposits, such as mixed-alluvial riverbeds it is unlikely that each substrate type is associated with a sufficiently narrow distribution of sidescan backscatter intensities to establish direct relations between sidescan backscatter intensity and substrate [36]. For finer substrates such as sand, this is due to large variations in slope, and bedform heights and wavelengths that collectively cause variations in backscattering strength. For coarser substrates such as cobbles, boulders and bedrock, the variation in backscattering of sound is caused by acoustic shadows that scale with both the height of the individual roughness elements but also the relative angle with the sonar [36]. Therefore, the majority of approaches to automated objective classification of substrates from echograms have used analyses of textural or variance properties of patterns that correspond to sedimentologically distinct regions [32, 34, 36, 38, 39]. An accurate substrate map might even provide a means with which to further correct echogram from which it is derived for radiometric distortions [37].

The word ‘texture’ has been used to describe variations in bed form morphologies [40], to map spatial arrangements of riverbed sediment [41], as synonymous with grain size distributions [11], and as a term to describe any surface roughness, rugosity, or waviness without strict definition [42]. The word is often used to substitute for a suite of variables related to the grain size, roughness, and the spatial arrangement of those quantities on the bed, where information on these quantities is lacking [43]. Roughness often refers to 1st-order metrics, such as the standard deviation of riverbed elevations [44], whereas texture often refers to 2nd-order metrics that take into occur the spatial arrangement of roughness elements or spatially continuous areas of like-roughness [43]. The word texture is often used in remote sensing in situations where the actual scale of interest, such as the bed form or grain scale, exist at the sub-pixel scale which is not resolvable but results in supra-pixel spatial arrangements of pixel intensities that indicate the presence and/or magnitude of the features of interest [45]. In a similar vein, here the word texture is used here in a qualitative sense to describe the spatial arrangement of surface roughness, that itself is the product of both supra-pixel (i.e. grouping of pixels) and sub-pixel (i.e. single pixel) grain size and morphologies, and in the quantitative sense as the value of a particular spatial statistic that is indicative of a particular band of grain sizes.

The objective of this study is to develop an unsupervised classification of substrates based on their corresponding textures in side scan echograms. This paper builds on Buscombe [22] by evaluating how echograms collected with a recreational-grade sonar system can be used to objectively identify classify and map (e.g. delineate) riverbed sediment. Perceptually homogeneous textures in an echogram are each characteristic of a different substrate, therefore discriminating among these textures using statistical techniques, creates a substrate map. We use a case study of multiple side scan sonar images a canyon riverbed to evaluate optimal texture metrics for broad-scale (a coverage of hundreds to thousands of square meters at a resolution of meters to decimeters) substrate classification. First, we examine textural characteristics of echograms from visually identified areas of interpreted substrate types. We then test and evaluate two classification approaches of differing complexity. Each classification approach is calibrated to a particular riverbed, before it is then applied to entire datasets from that riverbed in an unsupervised manner. We further evaluate the ability of the sidescan-derived substrate maps to reproduce reach-averaged proportions of substrates in calibrated acoustical substrate maps derived from multibeam sonar. Finally, we discuss how these methods could be applied to echograms of other mixed alluvial beds with a different sedimentary and morphological character, and the implications of using recreational-grade side sonar systems for characterizing riverbed sediment for physical benthic habitat assessment.

## Methods

This work was conducted with assistance from the U.S. Geological Survey Grand Canyon Monitoring and Research Center under research permits issued by the National Park Service. Our approach to building an unsupervised classification using statistical descriptors requires manual delineation of different substrates within the data. The manually delineated substrate classes serve as training zones to identify statistical descriptors that can discriminate substrate classes from each other. We then optimize the discriminatory power of a classification approach before applying the classification in an unsupervised way to larger portions of the data set. We then validate the classification by evaluating the unsupervised classification’s ability to classify manually delineated substrate classes.

### Data collection & study area

We collected side scan sonar data at a fish monitoring site spanning a 1.6-km canyon-bound reach of the Colorado River [46–48]. The study reach is located 98-km downstream of Lees Ferry in Marble Canyon, Arizona, directly upstream from the confluence of the Little Colorado River, and covers multiple pool-riffle sequences. Data were collected during five river trips between May 2012 and April 2015 (Table 1) between fish sampling activities during quarterly fish sampling trips by various operators and boatmen, who had little or no prior knowledge collecting these data. Data were not quality-controlled in the field, and no repeat surveys were conducted. This protocol was intentionally designed to mimic rapid, opportunistic sampling. At a minimum, data were collected over the entire fish sampling reach, but trip-by-trip survey extents were dictated by the availability of operators and the logistical requirements of the fish sampling activities.

**Table 1:** Echogram inventory.

Trip Date	Number of Scans	Number of Usable Scans
05/2012	5	0
04/2014	6	4
05/2014	8	2
09/2014	10	6
04/2015	6	6

Additionally, Grams et al. [49] extensively mapped this study reach with multibeam sonar. The multibeam sonar data provide high resolution bathymetry for validating the positional accuracy of georectfied echograms, and independently derived sediment classification maps derived from the recorded acoustic backscatter [50] for evaluating side scan sonar sediment classifications. The riverbed of the study reach is well studied [36, 41, 50, 51], composed of non-cohesive sediment, with grain size ranging from fine sand to boulders, and containing no submerged vegetation.

Continuous side scan sonar recordings and positional information were collected with a Humminbird ^®^ 998c recreational-grade side sonar. The sonar was mounted to a pole off the starboard bow or abeam to starboard of a small (2.75-m long) aluminum-hulled boat with an outboard motor. Positional errors associated with poor GPS fix, and the lack of boat attitude information, were significant in this canyon setting with limited visibility of satellite constellations and areas with swift moving water. However, by collecting data in the middle of the channel and at low speeds the effects of canyon walls, boat pitch, heave and dynamic draft were minimized. The boat operator avoided crabbing, to ensure that the direction of progress best estimated the boat’s true heading, by either motoring with, or directly against, the main current.

### Sonar data processing

All of the echograms analyzed in this paper were processed using PyHum [22], an open-source toolbox for decoding the file formats associated with side scan sonar recordings from a Humminbird^®^ Side Imaging Systems. PyHum is a python-based, modular toolbox that currently supports multiple models from the HD-SI (i.e. 700, 800, 900 and 1100 series), HELIX, MEGA, and ONIX side imaging systems. This study used a HD-SI 998c. The data collected within this study were processed using the ‘read’, ‘correct’, ‘remove shadows’, and ‘map’ modules. Continuous side scan recordings are encoded in proprietary file formats that consist of a DAT file and set of SON files. Using the meta-data encoded within DAT file, the read module decodes the raw data contained in the SON files to produce a time series of data (i.e. scan lines) that represent the ensonified water column and sediment-water interface. The timing and strength of each ping within a scan line are then reoriented using a simplified sonar geometry model to establish the most likely longitudinal orientation of returned signals. The scan lines collected during a continuous recording are compiled into an echogram using the positional data recorded by the GPS antenna. The correct module applies basic geometric and radiometric corrections to account for the effects of environmental conditions (e.g. sound absorption) and sonar settings (e.g. signal strength and beam pattern). The remove shadows module was used to visually segment and remove areas devoid of texture (i.e. water column and acoustic shadows) that exist in the near-field and far-fields of an echogram. The map module was then used to project the corrected and filtered echogram to a known coordinate system using the positional and navigational information collected with the supplied GPS antenna in the units of decibel watts (dBW). Buscombe [22] has detailed the data processing assumptions and acoustic corrections encoded within the software. PyHum differs from other available recreational-grade side scan sonar processing software [52, 53] because it radiometrically corrects the backscatter data, and projects each pixel in the echogram as a point in a point cloud using instantaneous position and heading, rather than rubbersheeting the raster using image rectification, which can lead to greater positional errors.

The resulting georeferenced side scan intensity points clouds are very spatially dense, with up to thousands of points per square meter. When resampling large point clouds consisting of millions of points, [43] found that a nearest-neighbor approach using a K-dimensional (K-D tree) was the fastest algorithm, even less computationally expensive than computing a mean of all points in the cell. This is because the former involves only two operations per grid node: 1) finding the nearest points to the node and 2) ascribes its value to the node. The mean (or other summary statistic) involves three operations: 1) finding the points, 2) computing the mean, and, 3) ascribing the mean to the node. The PyHum program does in fact offer three different ways to resample the data: 1) nearest neighbor (the approach we used for this manuscript); 2) inverse distance weighting, which is an average of nearest neighbors weighted inversely according to distance from grid node; and 3) average of nearest neighbors weighted by a Gaussian kernel. The latter two approaches often result in less noisy grids, but are significantly slower. The resampled side scan intensity points clouds were then converted to a raster format in Arizona Central State Plane, NAD83. The grid size 0.25 x 0.25 m was chosen to ensure each cell had multiple data points. The side scan intensity images were then processed outside of PyHum, using a program written by the authors, to derive the textural properties identified in this paper.

### Visually identified sediment patches

**Fig 1:**
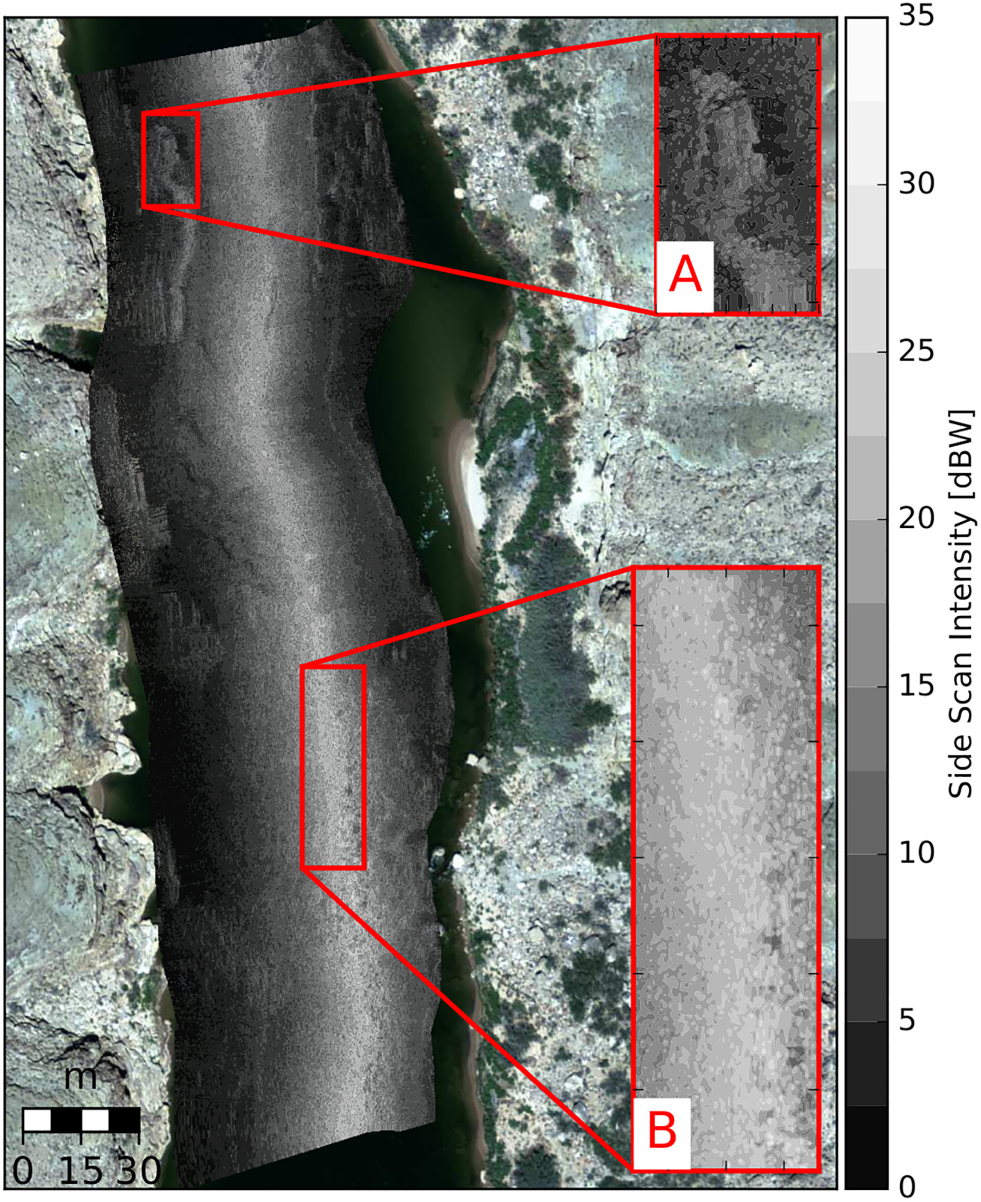
An example of georectfied echogram. A: indicated an apparent morphology that was ignored during visual delineation. B: is an example of an over-saturated region where the textural signatures are difficult to interpret.

Echograms consist of 8-bit digital integers representing the backscattering strength of the bed, called ‘grey levels’. The georectfied side scan sonar images were used for the visual classification of substrates into broad Wentworth-style groupings of similar sediment to develop calibration and validation data sets. Based on the quality of the available data (Table 1), visual delineation of echograms into three sediment classes was deemed appropriate to establish a data set to evaluate pixel-by-pixel classification by automated analyses. At least three distinct substrate types could always be reliably be distinguished. The textures shown within the echogram are created by sedimentary and morphologic features. Smooth (i.e. low contrast), highly ordered textures were associated with mixtures of sand. Rough, disorderly textures are associated with boulders and bolder-dominant mixtures of gravel/boulders and sand/boulders. Textures that vary between smooth, orderly and rough, and disorderly were associated with gravel and gravel-dominant mixtures of sand/gravel and gravel/boulders. Hereafter, these classes are referred to as sand, boulders, and gravel, respectively. The visual delineation was carried out in a Geographic Information System at a fixed scale of 1:600. Over-saturated regions of the echogram and apparent morphologic features were excluded from the delineation (Fig 1). These over-saturated regions are portions of the echogram directly beneath the boats track line where the first (nadir) returns are so much greater in intensity than subsequent returns that the 8-bit quantization is insufficient to capture the full dynamic range of backscattered sound.

### Texture metrics

#### First-order statistics

First-order statistical signatures of sediment types were developed using zonal statistics calculated from the visually mapped substrate patches and georectfied echograms. Both statistics of central tendency (i.e. mean, quartiles) and statistics that describe the distributions shape (i.e. standard deviation (*σ*), coefficient of variation (CV), kurtosis (*γ*), and skewness) were considered.

#### GLCM texture metrics

The Grey Level Co-Occurrence Matrix (GLCM) is a second-order (i.e. quantifying spatial relationships) statistical method and has been found suitable to describe textures in echograms [31, 32] because it statistically describes spatial relationships between pixels within a local area, and because a number of objective measures can be computed from it. A GLCM is a matrix within which the frequency of tonal patterns between pixel pairs within a computational window are tabulated [54]. For a reference pixel in a computational window of size *L* × *L*, a GLCM is calculated by specifying a reference angle *θ*, distance *d* and number of gray levels *N* to quantize the original image. Values within a co-occurrence matrix are typically normalized such that the values represent a probability rather than a frequency of a particular pixel-pair relationship [55]. A GLCM (*P*_(*i*, *j*)_) for a given computational window:

$$\begin{array}{c} P_{\left(i,j\right)}=\frac{V_{\left(i,j\right)}}{\sum_{i,j=0}^{N-1}V_{\left(i,j\right)}} \end{array}$$ (1)

where *V*_(*i*, *j*)_ is the co-occurrence matrix and *i*, *j* are reference and neighboring pixel values, respectively. If a value in a GLCM is large, the specific tonal pattern it represents is common and associated with textures within the computational window that are repetitive. When a value in a GLCM is low that specific tonal pattern is uncommon and textures within that computational window are random. Orderly, repetitive patterns of grey levels are interpreted as being created by features which exist at the pixel (25 cm) or sub-pixel scale and are interpreted as finer substrates (i.e. sand), whereas disorderly patterns of gray levels are created by supra-pixel scale features and are interpreted as coarser substrates (i.e. coarse gravel, boulders) [32].

Haralick [54] proposed 14 scalar metrics for description of textural patterns encoded in a GLCM. These properties are amenable to a spatially explicit analysis, whereby each scalar coefficient is computed from each co-occurrence matrix and assigned to the computational window it represents. The Haralick texture descriptors have been shown to be applicable to vary a wide spectrum of natural and artificial textures, but each can be thought of as belonging to one of three groups: namely, contrast, orderliness, and descriptive statistics [55]. Blondel [32] was the first to identify Entropy (group: orderliness) and Homogeneity (group: contrast) properties as useful for classifying echogram textures. They are defined as, respectively:

$$\begin{array}{c} H=\sum_{i,j=0}^{N-1}\frac{P_{\left(i,j\right)}}{1+{\left(i-j\right)}^{2}} \end{array}$$ (2)

$$\begin{array}{c} E=-\sum_{i,j=1}^{N-1}P_{i,j}\ln\left(P_{\left(i,j\right)}\right) \end{array}$$ (3)

Blondel et al. [33] further suggested that if *E* and *H* have a strong negative correlation the end members of such a relationship represent boulders and sand, respectively. *H* is a useful indicator of image contrast because the term (*i* − *j*)^2^ eliminates the diagonal terms of a co-occurrence matrix and therefore is weighted using only of the off-diagonal (i.e. *i* ≠ *j*) matrix elements. Therefore, highly contrasted textures produce low *H* values and textures with low contrast are characterized by high values of *H*. Entropy characterizes the orderly components of a GLCM. Large values of *E* occur when there is a wide distribution of grey levels.

Through a process of elimination and evaluation of the remaining 12 Haralick metrics, we found GLCM variance (group: descriptive statistics) to be another potentially useful GLCM property for sediment discrimination. GLCM variance ($\sigma_{G}^{2}$) and *E* are related, since they both quantify the dispersion of differences in intensity between pixel pairs, but GLCM variance is a statistic computed from the GLCM itself, given by:

$$\begin{array}{c} \sigma_{G}^{2}=\sum_{i,j=0}^{N-1}{\left(i-\mu \right)}^{2}P_{\left(i,j\right)} \end{array}$$ (4)

where GLCM mean is:

$$\begin{array}{c} \mu =\sum_{i,j=0}^{N-1}iP_{\left(i,j\right)}. \end{array}$$ (5)

A sliding 2D window approach was used to calculate GLCMs over small regions of the image. Neighboring windows had no overlap in either direction and a GLCM was only computed when at least 75% of the window contained data. To determine how various GLCM calculation parameters affect echogram texture segmentations, GLCMs were calculated with a parameter space with varying window sizes, search distances, and reference angles (Table 2).

**Table 2:** Variables tested for GLCM calculations.

Variable	Parameters
Search Distance (*d*) (pixels)	1, 5, 8
Reference Angle (*θ*) (°)	0, 45, 90, 135
Window Size (*L* × *L*) (m)	2, 3, 5, 10, 20
GLCM Properties	Entropy, Homogeneity, GLCM mean, GLCM variance, GLCM correlation

### Texture segmentation and sediment classification

#### Linear least-squares

A linear least-squares classification was developed to classify sediment into *q* sediment types using *N* classifying vectors *V* consisting of statistical measures of image texture. The process of assigning a scalar value to each sediment type results in the loss of a significant amount of information because each sediment type represents sediment of various sizes and is best described by a distribution of values. The proportion of variance explained by each of *q* sediment type is:

$$u_{q}=\min\underset{o}{|}|(uo)+C|{|}^{2}$$ (6)

where:

$$\begin{array}{c} C=[\bar{\left(V_{1}\left(1:q\right)\right)},\ldots ,\bar{\left(V_{N}\left(1:q\right)\right)}] \end{array}$$ (7)

$$\begin{array}{c} o=[\left(V_{1}\left(1:n\right)\right),\ldots ,\left(V_{N}\left(1:n\right)\right)]. \end{array}$$ (8)

The ∥ indicates an Euclidian norm. The resulting probability of each sediment type is estimated using [50]:

$$\begin{array}{c} \alpha_{q}=u_{q}\sum_{n\subset q\neq n}\left(1-u_{n}\right). \end{array}$$ (9)

Since ∑ *u*_*q*_ = 1, *α*_*q*_ = 1 for a particular sediment type indicates zero confidence in all other sediment types and we therefore have complete confidence in that particular sediment type. In the unusual case where for *n* sediment types *u*_*q*_ ≈ 1/*n* for all *n* sediment types, a classification is considered indeterminate because equal confidence would exist in all sediment types. After the model is calibrated, a weighting (*w*_*q*_) can be applied to each variable to produce the highest classification accuracy. Optimal weightings were identified using an optimization technique where the weights were evaluated in increments of 0.1 and constrained such that ∑ *u*_*q*_ ∑ *w*_*q*_ = 1. In the situation where all sediment types had low confidence (i.e. 0.15 < *α*_*q*_ < 0.25) the classification was considered indeterminate and is assigned a null sediment type [50]. Representing a substrate by the mean of an associated texture metric is simplistic, however this linear least-squares classification approach allows us to determine the viability and parsimony of more sophisticated classification approaches.

#### Gaussian Mixture Model

With an expectation that there exists a distribution of each texture metric associated with each substrate type, we considered a Gaussian Mixture Model (GMM) approach to classification. A GMM, which has been used in a recent study [56] to classify riverbed substrates from populations of multibeam backscatter, is a model for non-normal distribution as a a mixture of continuous distributions consisting of a finite number of Gaussian density functions [57]. Each Gaussian density function in this case represents a distribution of texture values from a given metric associated with a discrete substrate class.

A GMM is a weighted sum of *q* components (substrates) within a distribution of any suitable texture measure, *v*, expressed as

$$\begin{array}{c} p\left(x\right|\lambda )=\sum_{x=1}^{q}w_{x}N\left(v\right|\mu_{x},\Sigma_{x}) \end{array}$$ (10)

subject to:

$$\begin{array}{c} \sum_{x=1}^{q}w_{x}=1 \end{array}$$ (11)

where $N(\mu_{x},\Sigma_{x})$ is an individual Gaussian density function, described by covariance matrix Σ_*x*_ and weightings assigned to each model component, *w*_*k*_, and calculated as:

$$\begin{array}{c} N\left(\mu_{x},\Sigma_{x}\right)=\frac{1}{{\left(2\pi \right)}^{D/2}}\frac{1}{{\left|\Sigma \right|}^{1/2}}\exp\left\{-\frac{1}{2}{\left(x-\mu \right)}^{T}\Sigma^{-1}\left(x-\mu \right)\right\} \end{array}$$ (12)

where *μ*_*x*_ is the mean vector of the *X*, *D* is the dimension of the vector *X*, and *E*[(*x* − *μ*)^*T*^ (*x* − *μ*)] is the covariance matrix. The model parameters, λ = [*μ*_*x*_, Σ_*x*_, *w*_*x*_], are estimated using the Expectation-Maximization (E-M) algorithm [58]. The likelihood of the model given the training data is maximized by iteratively evaluating candidate parameters λ. The conditional probability of the sequence of *T* training vectors *V* = [*v_i_*, …, *v_T_*] given a parameter set, λ, is

$$\begin{array}{c} p\left(V\right|\lambda )=\prod_{t=1}^{T}p\left(v_{t}\right|\lambda ). \end{array}$$ (13)

Beginning with an initial proposed λ (typically GMMs are initialized by estimating the mean and variance of *V* and unit weighting), a new model λ′ is proposed and accepted if *p*(*V*|λ′) > *p*(*V*|λ). This process is repeated until the E-M algorithm converges on the solution that best represents the data. The Expectation step involves assigning data points to Gaussian density functions by maximizing the likelihood probability a data point came from a particular distribution. Current λ is used to estimate posterior probability, given by

$$\begin{array}{c} P\left(i\right|v)=\frac{w_{x}g\left(v_{t}\right|\mu_{x},\Sigma_{x})}{\sum_{x=1}^{q}w_{q}N\left(v_{t}\right|\mu_{q},\Sigma_{q})} \end{array}$$ (14)

The Maximization step is where λ′ is re-estimated using the probabilities calculated during the E-step. Since posterior probabilities are computed per-pixel and per-substrate, they offer a ready means with which to evaluate classification uncertainty in a spatially distributed sense, or define acceptance criteria for a given classification.

We considered several covariance models, including ‘full’ ($\Sigma =\frac{1}{q-1}\sum_{x=1}^{q}\left(v_{x}-\mu_{x}\right){\left(v_{x}-\mu_{x}\right)}^{T}$), constrained to be diagonal ($\Sigma =\frac{1}{q-1}\sum_{x=1}^{q}{\left(v_{x}-\mu_{x}\right)}^{2}$), or spherical (symmetrical in all directions, or $\Sigma =\frac{1}{D(q-1)}\sum_{x=1}^{q}{∥v_{x}-\mu_{x}∥}^{2}$, where *D* is the number of model parameters). Additionally, we considered a common covariance matrix for all *q* component substrates, termed a ‘tied’ covariance model where a full covariance matrix is shared among all of the Gaussian density functions. To determine the optimal number of substrates and form of the covariance model, an optimization was performed using the Bayesian Information Criterion (BIC, [59]) as a cost function. BIC scores are used to identify a best fitting model with the lowest number of model components. Models with too many components are prone to over-fitting the data and are assigned a higher BIC score than models with fewer components. Similarly model with too few components under-fit the data and are assigned higher BIC scores than models with more components. Thus, the optimal value of *q* and covariance model that collectively resulted in the lowest BIC score.

#### Substrate classification skill

Each unsupervised classification algorithm was evaluated using accuracy (true positives) as well as precision and recall metrics that are commonly used to accounting for Type 1 (false positive) and Type 2 (false negative) errors. An *F*_1_ score is a weighted average of precision and recall, taking values between 0 and 1, and is given by

$$\begin{array}{c} F_{1}=2(\frac{PR}{P+R}) \end{array}$$ (15)

where precision, *P*, is the number of true positives in the classification divided by the sum of true and false positives, and recall, *R*, is the number of true positives divided by the sum of true positives and false negatives.

## Results

### Sediment texture signatures

In the following subsections, the utility of first and second order (GLCM) statistics are evaluated to identify objective metrics that could be used for the development of automated pixel-by-pixel sediment classification algorithms.

#### First-order statistics

For each substrate type identified with visual mapping, the underlying distributions of side scan intensity values were aggregated before we calculated summary statistics (Table 3).

**Table 3:** Aggregated side scan intensity distribution summary statistics.

Substrate	Mean	*σ*	CV	25%	50%	75%	Kurt	*γ*	n
Sand	8.401	3.572	2.227	5.484	8.772	11.187	−0.662	−0.118	71
Gravel	5.531	3.721	2.243	5.996	8.601	11.137	−0.522	−0.05	30
Boulders	8.702	4.422	1.976	5.261	8.464	11.89	−0.643	0.036	18

**Fig 2:**
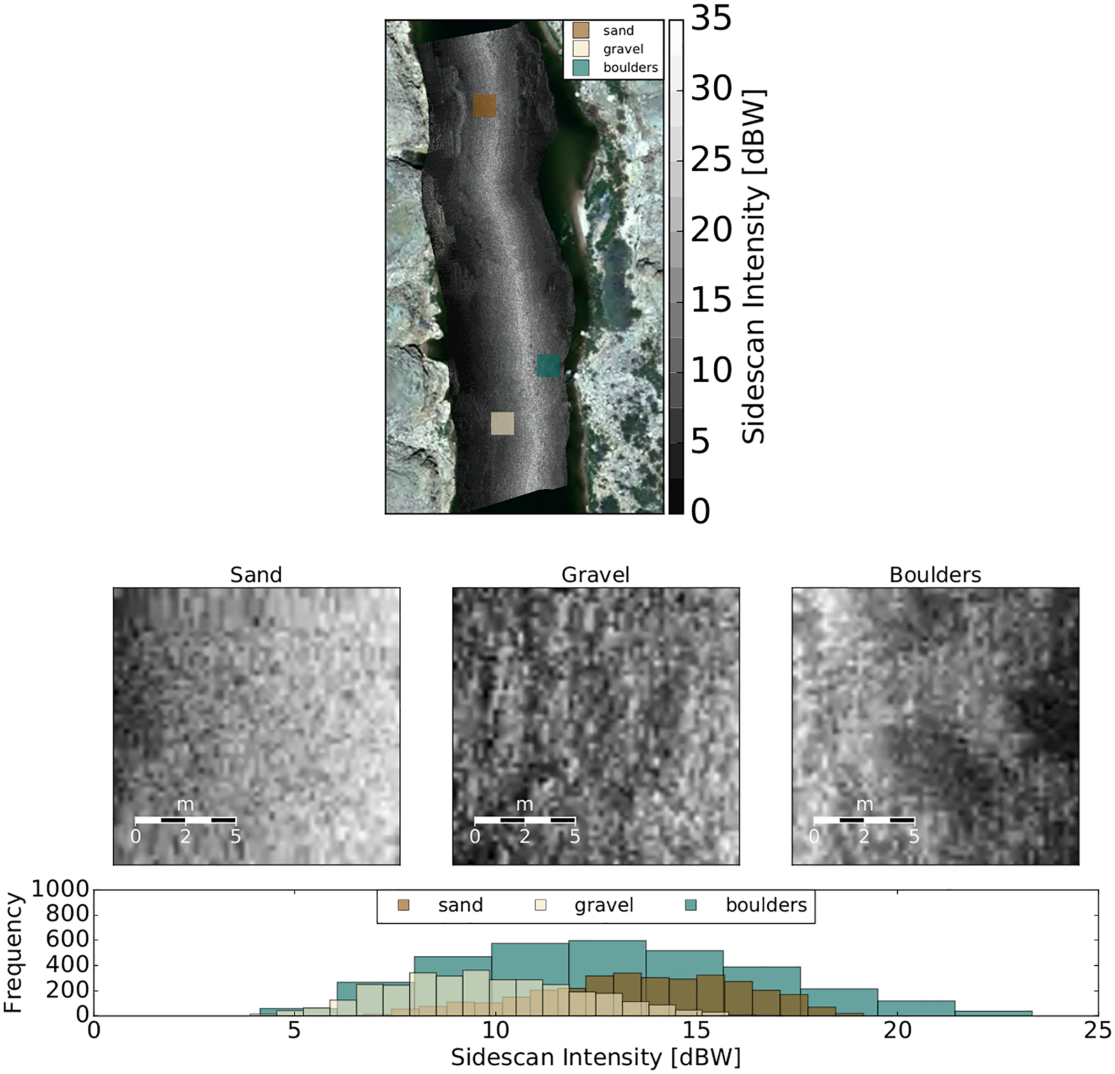
Example of side scan sonar intensity distributions of three visually identified sediment types.

Statistics describing the magnitudes of aggregated side scan intensity distributions are of limited use for sediment discrimination because there is a high degree of overlap between sediment types (Fig 2). Standard deviation is potentially more useful because it increases with grain size. However, when the thresholds between sediment type and standard deviation (Table 3) are tested using varying window sizes, the relationship proves to be inconsistent.

#### GLCM texture metrics

In total, 300 texture features were evaluated to determine which combination of GLCM parameters could be used to most reliably discriminate between sediment types. An ideal combination of GLCM parameters results in texture distributions that each have significantly different means and small variances. The textures associated with sand and boulders are captured in the tails of the distributions, while the textures associated with gravel separate them from each other.

**Fig 3:**
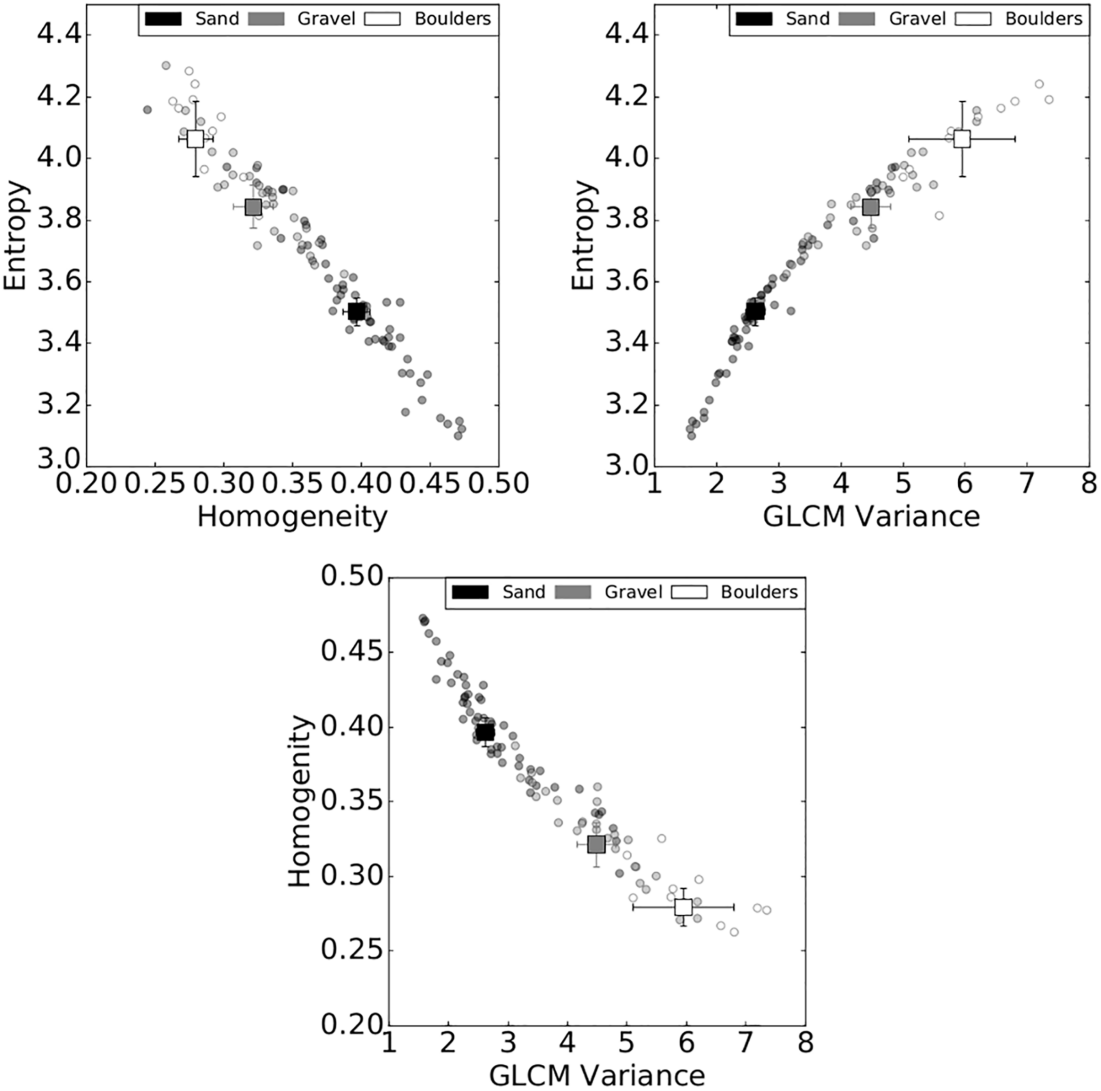
Bivariate analyses of Entropy, Homogeneity, and GLCM variance. Circle data points indicate median values for each of the visually identified substrate paths. Square data points are the bootstrapped median values with error bars indicating the 95% confidence interval. GLCMs were calculated using a search distance of *d* = 5, reference angle of *θ* = 0°, and a window size of 9 m^2^.

In agreement with previous studies [31–33], Entropy (*E*) and Homogeneity (*H*) were identified as particularly sensitive to substrate type. GLCM calculations were sensitive to computational window size. Smaller window sizes (i.e. <10 m) captured the textural variations of the echograms and produced texture features with wide distributions. A window width of *L* = 3 m (a window size of 9 m^2^) best captured the textural variations and produced wide distributions of *E* and *H*. Of the three search distances evaluated, *d* = 5 pixels (i.e. 1.25 m) best captured the textural variations of georeferenced echograms. Search distance *d* = 1 resulted in a wide distribution of *H* with narrow distributions of *E*, while *d* = 8 resulted in wide distributions of *E* and narrow distributions of *H*. Reference angle had little effect on the distributions of *E* and *H* and we therefore set the reference angle to *θ* = 0. Of the other GLCM statistical properties, GLCM mean had a weak correlation between sediment type and produced the lowest amount of clustering, whereas GLCM variance, $\sigma_{G}^{2}$, was found to have a much stronger relationship with sediment type (Fig 3).

### Broad-scale sediment classification models

**Fig 4:**
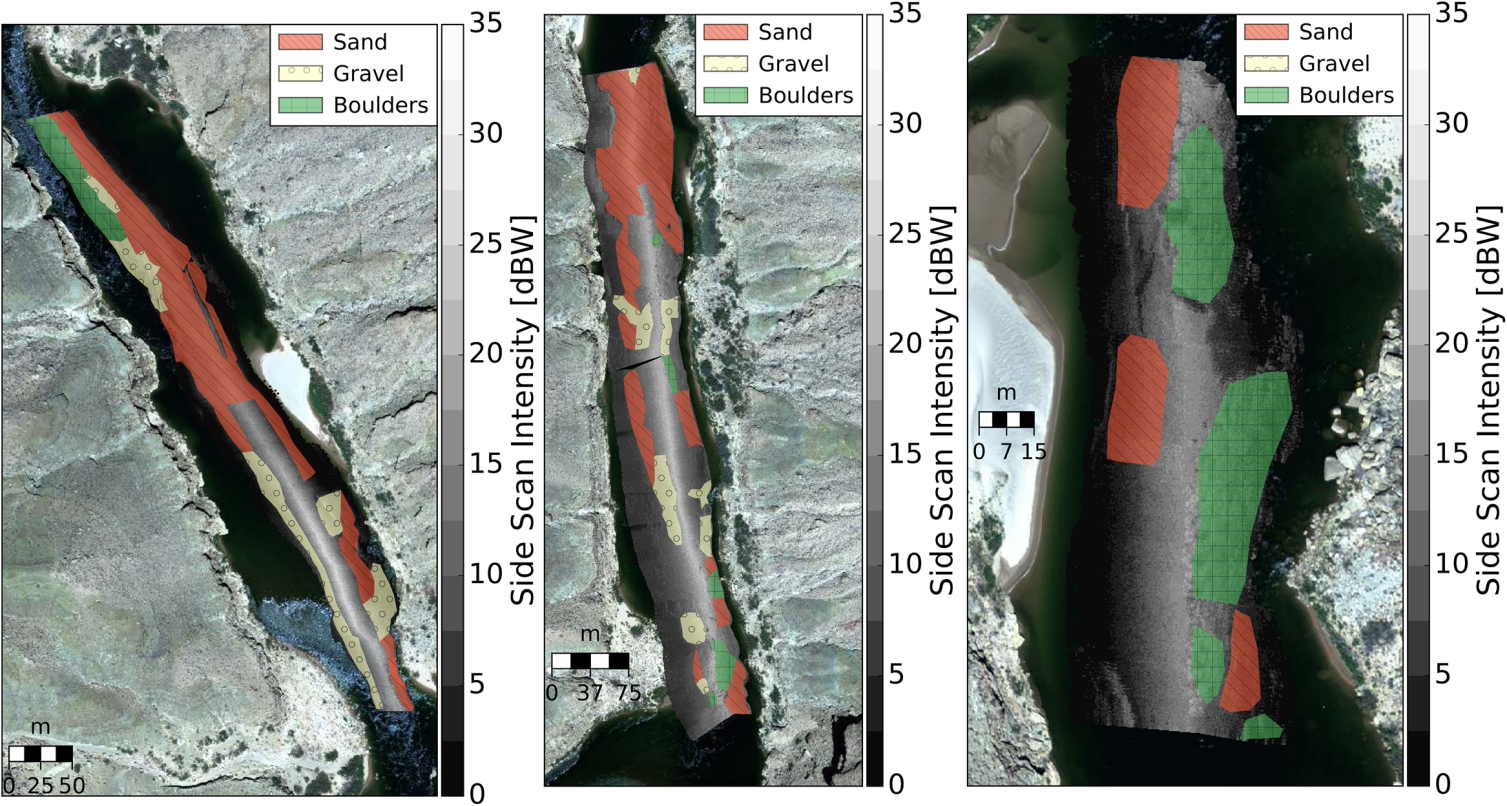
Visually identified patches used to validate substrate classifications. A total of 71, 30, and 18 patches were visually identified substrates sand, gravel, and boulders, respectively. All patches were digitized at a fixed scale of 1:600. Sand, gravel and boulders average polygon sizes of 583, 306, and 334 m^2^.

**Fig 5:**
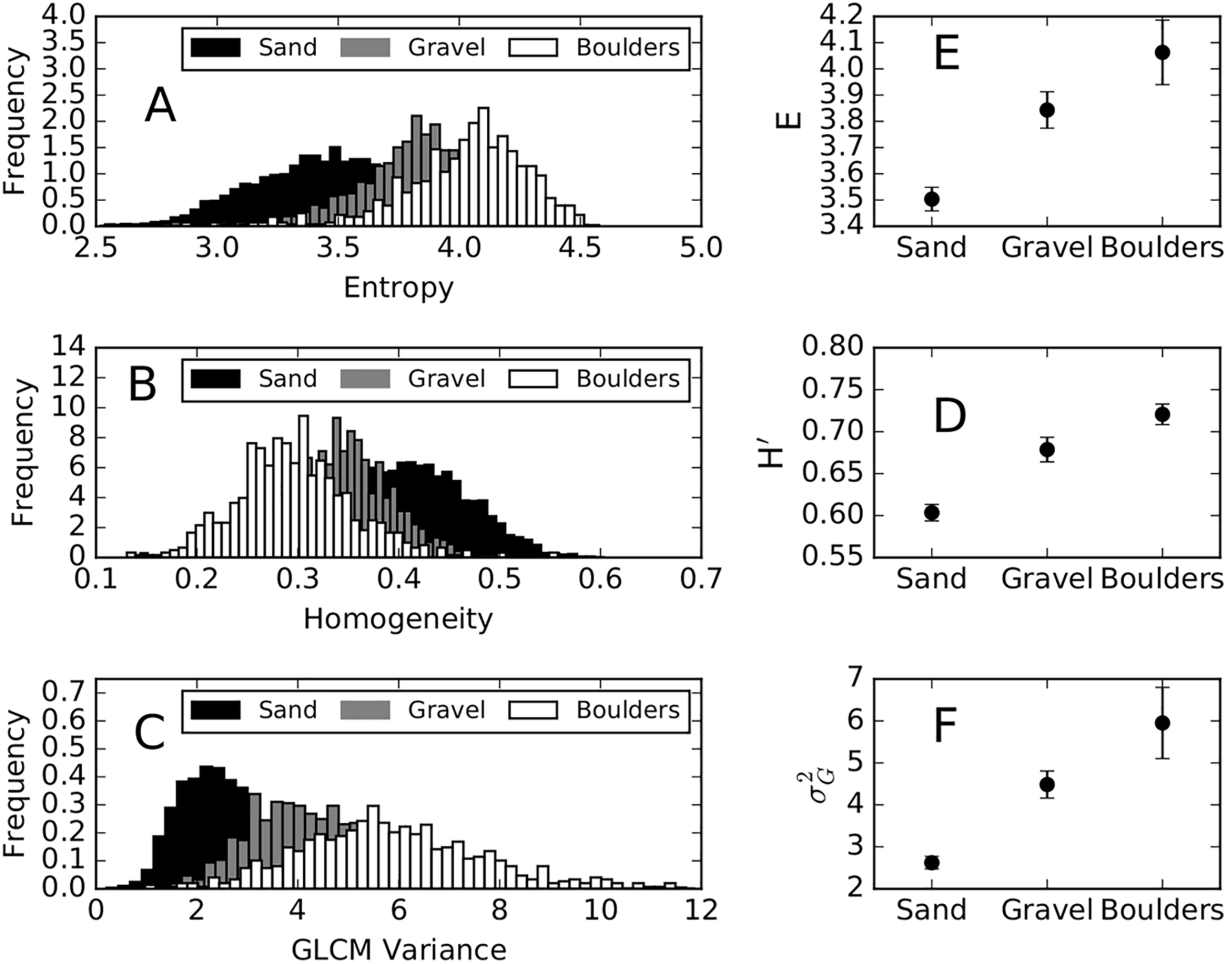
Panels a through c: Aggregated distributions of GLCM texture features Entropy, Homogeneity, and GLCM variance. GLCM texture features were calculated using search distance *d* = 5, reference angle *θ* = 0°, and window size 9 m^2^. Panels d through f: linear least-squares classification data for model calibration. Data points indicate median values and error bars indicate 95% confidence intervals. The relationship between Homogeneity and sediment size shown in Fig 3 was reversed using Eq 16.

For the purpose of broad-scale (1 classification per 9 m^2^ of riverbed) substrate characterization into 3 sediment types, the two sediment classification techniques were developed and tested. The methods were evaluated, using the metrics described in this paper, based on their ability to correctly estimate sediment types within the visually mapped patches (Fig 4). The aggregated distributions of each textural feature (Fig 5a–5c) are non-normal in shape, but the individual sediment types are unimodal and approximate normal distributions.

#### Linear least squares

A linear least-squares model was developed using *H*, *E*, and $\sigma_{G}^{2}$. Since *E* and $\sigma_{G}^{2}$ both have positively correlated relationships with grain size (Fig 3), the relationship between *H* and sediment size was made to conform to the same trend by using:

$$\begin{array}{c} H^{\prime }=1-H \end{array}$$ (16)

Initially, each sediment type was weighted equally and was used to develop a calibration matrix using median values calculated from a bootstrapping analysis with 10,000 samples (Fig 5d–5f). The median values calculated from a bootstrap analysis were used to calibrate the linear least-squares model. Bootstrapping was used to generalize the calibration so it could be applied to data collected in similar environments. The calibration matrix took the form $C=[\bar{\left(E(1:q)\right)},\bar{\left(H^{\prime }\left(1:q\right)\right)},\bar{\left(\sigma_{G}^{2}\left(1:q\right)\right)}]$, where *q* = 3 sediment types. The least-squares model (hereafter termed ‘LSQ’) was evaluated using the same visual substrate patches that were used to develop the sediment type calibration metrics (Fig 5d–5f).

Among the sediment types incorporated into the LSQ model, sand and boulders were classified with similar accuracy (Table 4). An optimized weighting of *w*_*q*_ = [0.1, 0.7, 0.2] was applied to the proportions of variance for each sediment type sand, gravel, and boulders, respectively to increase the overall classification accuracy for gravel. The weighting increased the gravel classification accuracy from 16% to 27.7% whereas sand and boulder classification accuracy changed from 85% to 75%, and from 70% to 72%, respectively.

**Table 4:** Least squares sediment classification confusion matrix.

	% Classified as‥		
Observed	Boulders	Gravel	Sand
Boulders	**72.6**	19.1	8.2
Gravel	46.2	**27.7**	25.9
Sand	10.5	14.0	**75.2**

#### Gaussian Mixture Model

The relative importance individual and combinations of texture metrics were evaluated by developing uninitialized GMM models for the spherical, diagonal, tied, and full covariance matrix types. The models were not initialized because any initialization could potentially result on the E-M algorithm converging on a locally optimal solution and therefore spuriously identify viable models. For each possible combination of texture features, a Bayesian Information Criterion (BIC) score was used to identify the number of model components (i.e. number of Gaussian density functions) and covariance model that produced the best fitting model.

The two optimal models were found to be: 1) a 2-substrate classification model using *E*, and 2) a 4 part classification model that combines *σ*_*G*_ and *H*′. Hereafter, the 2-part and 4-substrate GMM models are referred to as GMM-2 and GMM-4, respectively. GMM-2 can be used to identify sand and boulders only, whereas GMM-4 is considered to model sand, fine gravel, coarse gravel, and boulders. GMM-2 was initialized using the means associated with each sediment type. For GMM-4, estimates of mean values associated with fine gravel and coarse gravel were developed by interpolation between the known mean for gravel and the other two substrate types. The two gravel components within GMM-4 were both considered to represent a gravel classification during validation. Classification accuracy averaged across the three scans used during visual mapping (Fig 4) for GMM-2 and GMM-4 are presented in Table 5.

**Table 5:** Confusion matrices with average classification accuracy using the calibration data for GMM-2 and GMM-4.

	GMM-2 Classified as…			GMM-4 Classified as…		
	Sand	Boulders	Other	Sand	Gravel	Boulders
Sand	**85.9**		14.0	**60.7**	33.6	5.6
Gravel				15.6	**49.0**	35.4
Boulders		**85.9**	14.0	3.9	16.3	**79.8**

GMM-2 consistently produced very high accuracy when trying to classify sand and boulders (Table 5). This is mainly due the fact that the distributions of sand and boulders have minimal overlap compared to the distributions created by all three sediment types. GMM-4 produced a test accuracy of 59% and an average gravel classification of 49% (Table 5). Modeling gravel as two Gaussian distributions increased the overall proportion of correct gravel classifications, when compared to modeling it as a single Gaussian distribution.

**Fig 6:**
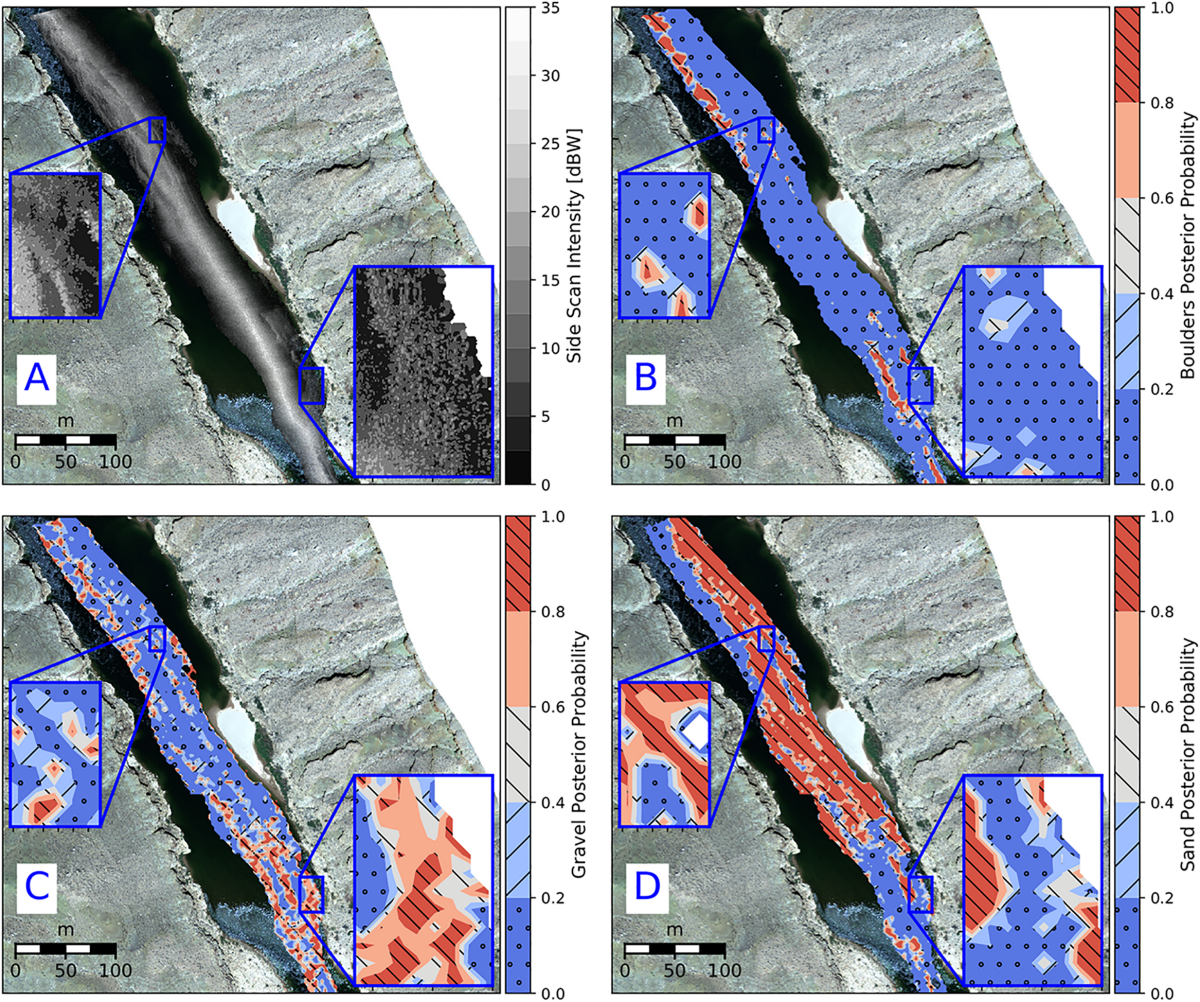
Comparison of the posterior probabilities assigned for each sediment class for the GMM-4 model. A: image depicting the boundaries between different textures. B: posterior probability map for boulders. C: Posterior probability map for gravel sediment class. D: Posterior probability map for the sediment class sand.

The classification accuracies (Table 5) are based on the maximum likelihood probability a 3×3-m pixel belongs to a particular Gaussian density function (i.e. sediment type). The spatial distributions of posterior probabilities for each sediment type offer a means to visualize each classification pixel’s membership among the modeled sediment types. To illustrate, the posterior probabilities assigned to each modeled component in GMM-4 for one of the echograms used to develop the model are presented in Fig 6.

### Model skill

Table 6 shows precision, recall and *F*_1_ scores for the LSQ, GMM-2, and GMM-4 models. The *F*_1_ scores for gravel are the lowest among all three sediment types, but indicate GMM-4 is produces more reliable gravel classifications. Among all three models, GMM-2 produces the highest *F*_1_ score is deemed the to perform the best.

**Table 6:** Classification mapping precision, recall and F1 scores for LSQ, GMM-2, and GMM-4.

	LSQ			GMM-2			GMM-4		
	*P*	*R*	*F*_1_	*P*	*R*	*F*_1_	*P*	*R*	*F*_1_
Sand	0.75	0.89	0.82	0.86	0.97	0.91	0.82	0.89	0.85
Gravel	0.28	0.31	0.29				0.56	0.38	0.46
Boulders	0.73	0.40	0.51	0.88	0.57	0.69	0.44	0.59	0.50
Average	0.66	0.66	0.64	0.86	0.86	0.85	0.71	0.72	0.71

### Substrate map comparisons

**Fig 7:**
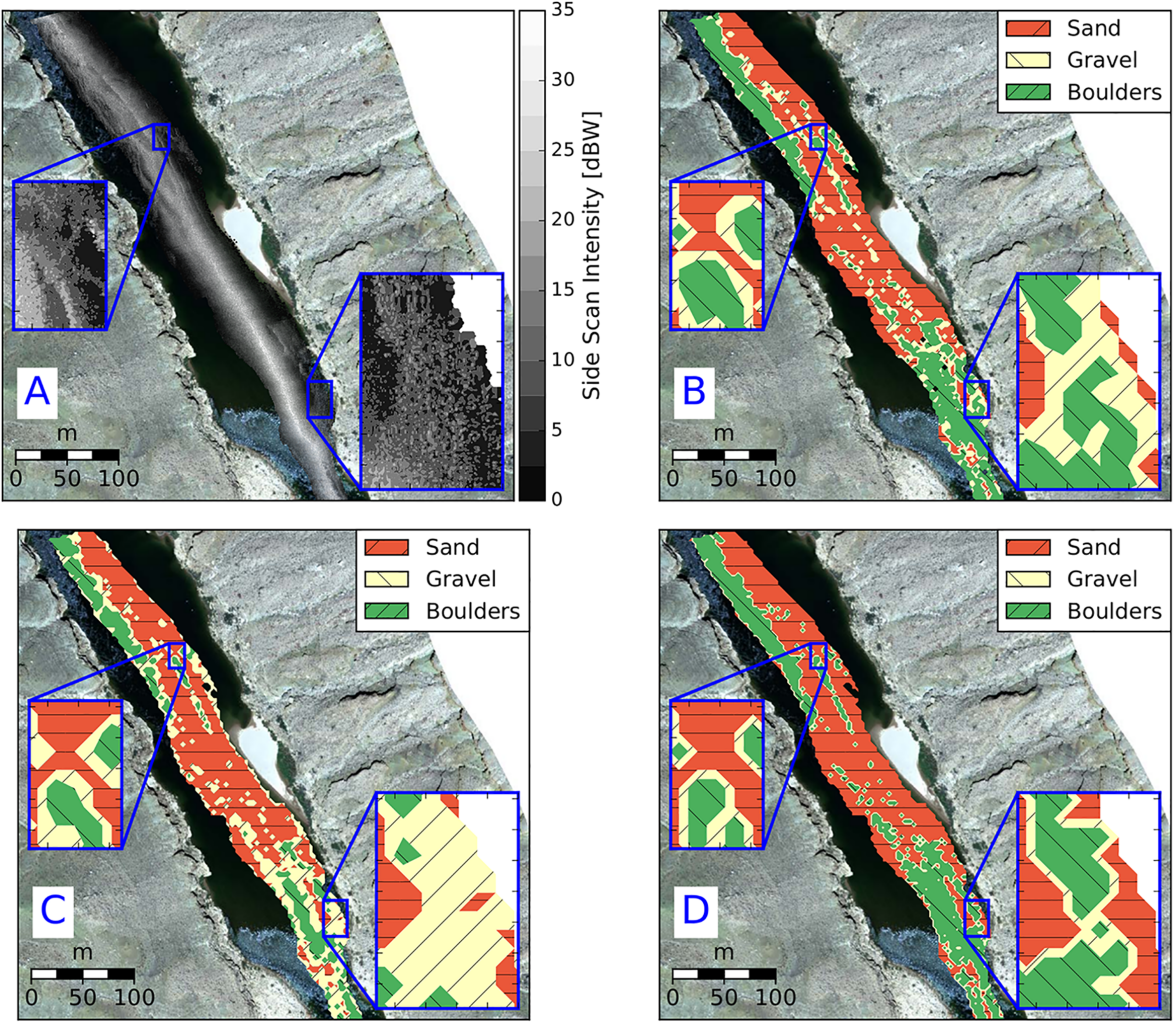
Comparison of sediment classification maps based on A) echograms, and contours of results from the B) LSQ, C) GMM-4, D) GMM-2 models. Water flows from top to the bottom of the image. Inset images show the boundaries between different textures.

The unsupervised sediment classification algorithms developed in this paper were used to develop a coarse-resolution (9 m^2^) sediment classification map (Fig 7) for one of the scans used during visual mapping (i.e. within-calibration). In a qualitative sense, all models produce similar spatial distributions of sediment types.

**Fig 8:**
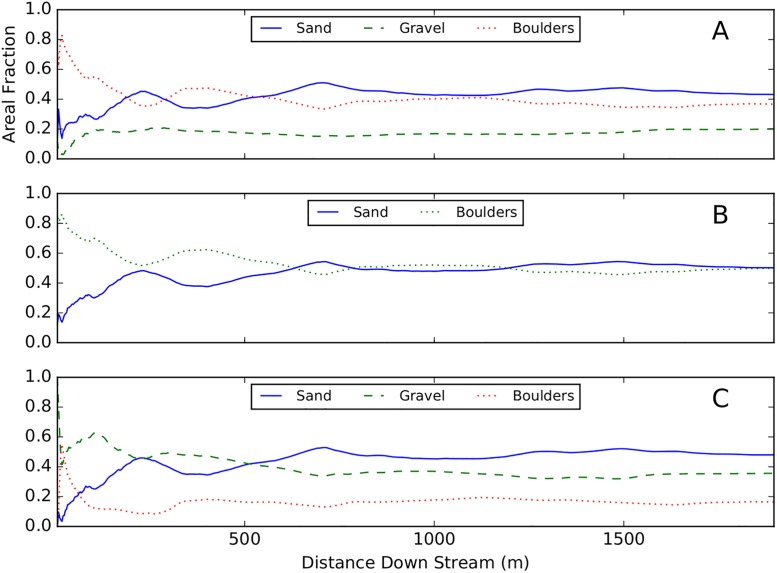
Cumulative areal fractions of each sediment type as a function of scan length. Subplots A, B, and C, correspond to the models LSQ, GMM-2, and GMM-4, respectively. The areal fractions of each sediment type equilibrate after ≈ 250 meters.

To determine the length of reach required to characterize the reach-averaged sediment proportions, substrate maps were computed using an echogram collected over the entire study area. Reach-averaged areal proportions were computed as a function of cumulative distance downstream (Fig 8). After ≈ 250-m downstream distance, the areal fractions of each sediment class converge to values characteristic of the reach. This is particularity encouraging for applying these models to large volumes of data, because it aids sample design, indicating ground-truth sampling efforts to calibrate GLCM-GMM or GLCM-LSQ models can be focused on a relatively small fraction of the reach.

### Out-of-calibration validation

We applied all three substrate classification models to other side scan data collected in similar sedimentary settings, to test the broader applicability. The LSQ, GMM-2, and GMM-4 models were applied to side scan sonar imagery collected at a rainbow trout monitoring reach approximately 48-km down stream from Lees Ferry, Arizona. Visually delineated areas of various substrates were used to evaluate model performance. The out-of-calibration reach is a relatively straight section of the Colorado River unaffected by debris fans [50]. Like the calibration reach, the riverbed is composed of non-cohesive sediment, and does not have any submerged aquatic vegetation. Unlike the calibration reach, the flow is not constricted by large debris fans, and therefore it has a very different hydraulic character.

**Fig 9:**
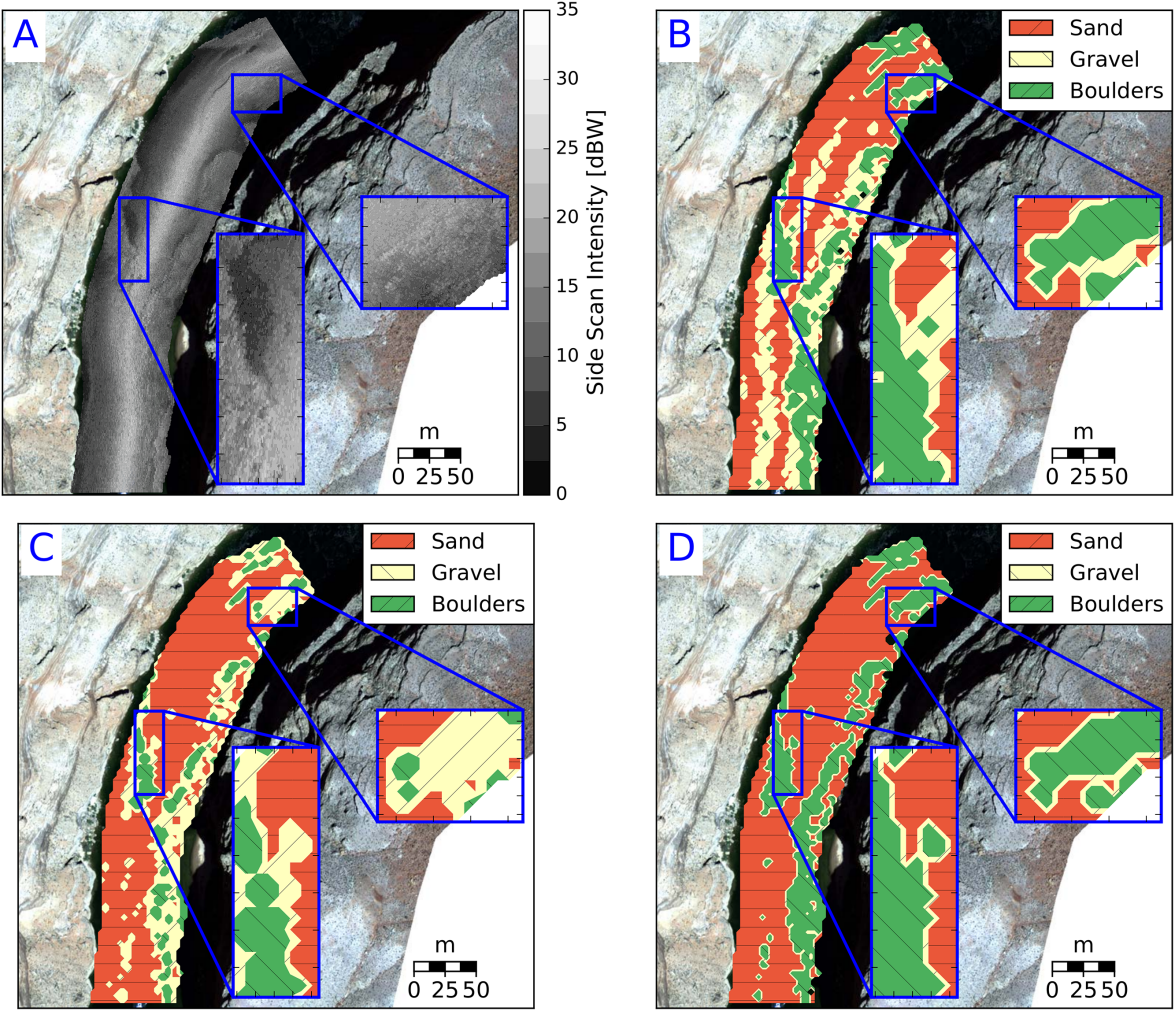
Comparison of sediment classification maps based on A: echograms, B: LSQ, C: GMM-4, D: GMM-2 at an out of calibration study reach. Water flows from top to the bottom of the image. Inset images show the boundaries between different textures.

All three models show promise for application in different sedimentary environments (Fig 9) and produced similar classification accuracy to the within-calibration data. Classification confusion matrices for LSQ, GMM-4 and GMM-2 are presented in Table 7. The primary difference between LSQ and GMM-4 is the presence/absence of gravel.

**Table 7:** Confusion matrices presenting acoustic sediment classification accuracy using out-of-calibration data for LSQ, GMM-2, and GMM-4.

	LSQ Classified as…			GMM-2 Classified as…			GMM-4 Classified as…		
	Sand	Gravel	Boulders	Sand	Boulders	Other	Sand	Gravel	Boulders
Sand	**59.4**	33.3	7.0	**95.0**		4.9	**31.3**	54.5	14.0
Gravel	29.3	**50.6**	19.7				5.4	**54.2**	40.2
Boulders	2.8	14.9	**82.3**		**82.3**	17.6	0.9	7.1	**91.9**

**Fig 10:**
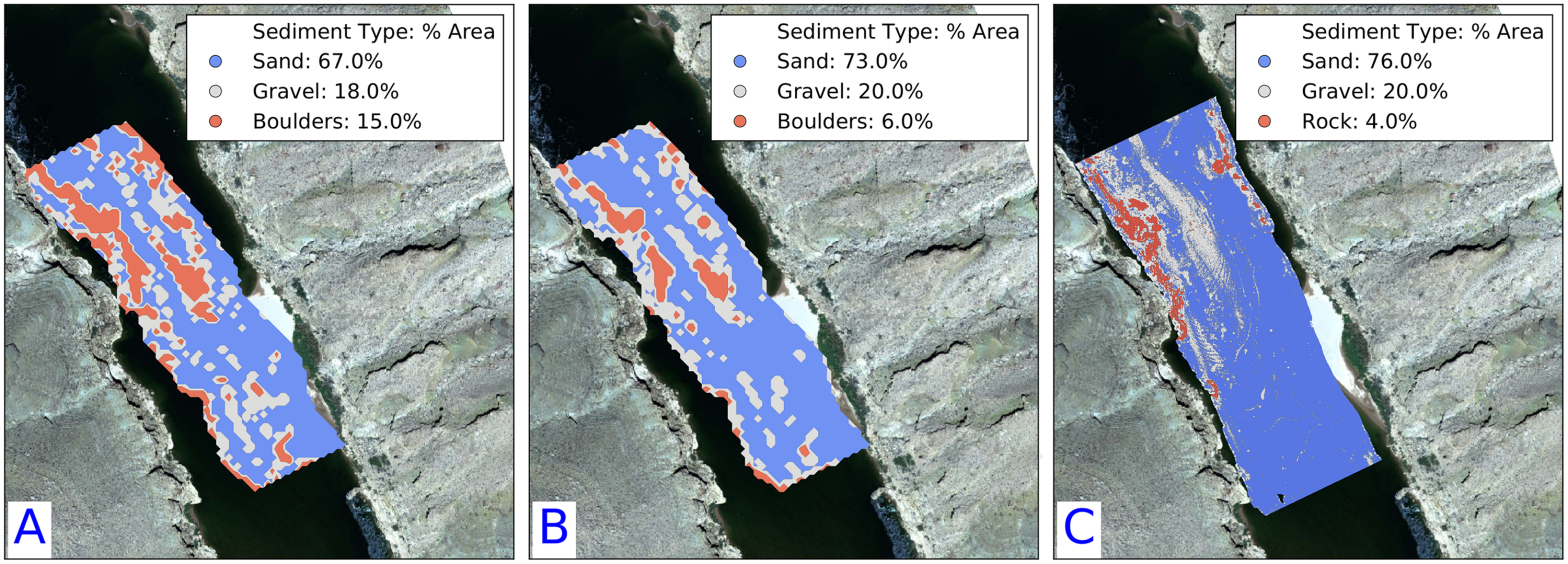
Comparison of sediment classification maps based on A: LSQ, B: GMM-4, C: Multibeam acoustic sediment classifications. Areal fractions of each sediment type are indicated in the legend. The spatial distributions of the sediment types are in disagreement between the models, but the reach averaged proportions of each sediment type are similar between all three models.

Both modeling approaches (LSQ and GMM) were compared to a multibeam sonar derived acoustic sediment classification developed by [50], also on a regular 25 cm grid (Fig 10). Reach-scale relative proportions of each sediment type are within a few percent, which suggests changes of bulk surface sediment redistribution through time are quantifiable. The spatial distributions sediment types are qualitatively similar (Fig 10) at the broadest scale, but there is significant pixel-by-pixel disagreement. We attribute this in part to poor GPS precision at the time of data collection. The positioning errors resulted in poorly positioned georeferenced echograms not only displaced it in the XY plane, but also resulted in the distortion of some of the echogram textures.

## Discussion

The approach we have outlined is designed for application to a specific range of grain sizes (sand, gravel, cobble, boulder and their mixtures). The calibration we have developed might be applied to similar substrates on other rivers. However, the methodology we have outlined here is transferable, consisting of three sequential steps, namely: i) manually identifying different textures within a data set that each correspond to a different substrate; ii) define statistical descriptors of those textures, and finally iii) use the classification based on these texture descriptors in an unsupervised sense to the entire data set. Following this procedure, we recommend that applications to other riverbeds, especially those with significantly different assemblages of bedforms and/or grain sizes to those here, or those with submerged vegetation, woody debris, or other organic matter, develop a site-specific calibration for optimal results.

### Substrate classification skill

In agreement with previous studies, we found that recreational-grade side scan sonar data has sufficient quality to derive statistical texture metrics that strongly relate to spatially varying bed sediment composition. Post-processing continuous recordings from using PyHum [22] allows the production of accurate side scan intensity point clouds that can be gridded for imaged based textural analyses.

Among the three sediment types modeled in GMM-4 and LSQ, gravel classifications vary the most. Poor classification rates for gravel is attributed to relatively poor bivariate clustering (Fig 3) and has significant amount overlap with the other sediment types. Gravel has a larger estimated areal proportion using GMM-4, being modeled as two components which better fit the larger continuum of textures associated with gravelly substrates. From a physical perspective, gravel classifications encompass grain sizes associated with the entire spectrum intermixed sand, gravel, and cobbles. Finer gravels create textures near or at the sub-pixel boundary and are similar to the textures created by sand, whereas coarser gravels create textures at the supra-pixel scale and are very similar to the textures associated with boulders.

The LSQ and GMM approaches each have their own merits for sediment classification using statistical texture metrics. In the LSQ approach, sediment types are characterized by the central tendencies of the distributions associated with sediment types. Ideally, a linear least-squares approach is best suited for a two part (i.e. sand and boulders) classification because the least-squares solution is biased towards distributions with minimal amounts of overlap. A GMM approach is desirable because it uses a probabilistic model for predicting sediment types from distributions of values, and because it assigns a posterior probability to each prediction, which can be used for quality control and uncertainty estimation. A GMM approach is better suited to higher order (i.e. >3 sediment types) classification problems because it allows sediment types to be described by a spectrum of textures.

### Window effects in textural segmentation

The necessity of, and process of, subjectively defining the window size for calculation of texture metrics using a traditional (square) windowed analyses has some limitations for texture analysis of echograms collected in a riverine environment. First, defining a grouping of pixels (i.e. a superpixel) that best captures the textural variations is highly dependent on the gridded resolution of the side scan sonar intensity point cloud, and the specific nature of the substrate, therefore is unlikely to universally applicable to any echogram. Second, constraining a computational window to be regular in size and shape imposes a constraint because riverbed sediment is not arranged in a regular way, therefore texture boundaries intersect grid cells and a given window may straddle a sharp sedimentary transition. Third, the depositional patterns or surficial riverbed sediment create sediment patches, whose area can vary by orders of magnitudes, and can therefore by represented by several computational windows. Therefore, adjacent windows with similar texture properties result in significantly increased computational cost because calculations are redundant.

**Fig 11:**
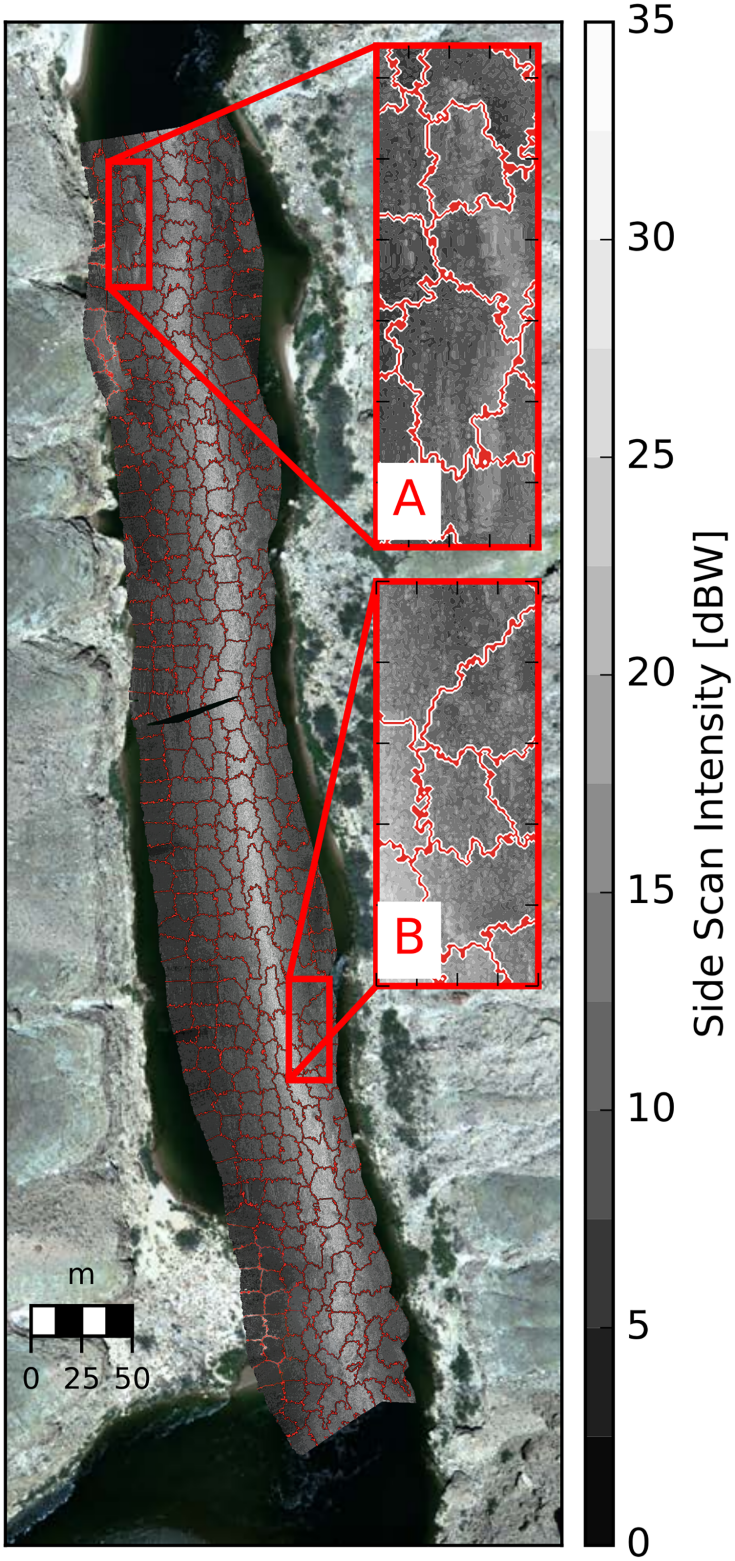
Example SLIC segmentation of a georectified echogram. There are 846 superpixels (as apposed to a 3x3 moving size, for example) that are delineated using red boundaries. The superpixels average area is 134 m^2^. A: shows a bed rock ledge where the SLIC algorithm failed and segments across the ledge face. B: shows a region where the algorithm performed well and accurately segmented a boulder field.

Simple Linear Iterative Clustering (SLIC) is an emerging segmentation algorithm in the field of computer vision [60] that has the potential to be applied to texture analysis of echograms and address the shortcomings of regular windowing. The SLIC algorithm automatically groups pixels based upon their textural variation and creates superpixels that are irregular in shape (Fig 11). In places where textural variations are minimal, the SLIC algorithm produces nearly rectangular superpixels. SLIC-based image segmentation can significantly reduce the number of calculations required for texture analysis because the resulting superpixels are significantly larger than the optimal window size identified in this paper. It allows for increased objectivity in the windowing procedure because a certain window size or shape need not be specified *a priori*. Finally, the SLIC algorithm can reliably identify sharp textural boundaries, therefore computed texture metrics in a given window may be more strongly associated with homogeneous patches of substrates.

The optimal number of superpixels for a echogram requires a subjective decision on the approximate number of superpixels required for the algorithm to capture the data boundaries of the georectfied echogram. Too few superpixels results in under-segmentation issues, such as 1) not segmenting between areas where textures are changing, and 2) creating super pixels around echogram boundaries which include significant amounts of the ‘no data’ region. Too many superpixels results in over-segmentation where regions of the echogram are segmented regardless of actual textural variations. An analysis of five echograms, whereby the number of superpixels was systematically varied, indicates that the number of super pixels required to segment the data boundaries increases approximately linearly with echogram length, and that texture varies at an average scale of 126 m^2^ (Table 8) for this study reach.

**Table 8:** Echogram SLIC superpixel parameters.

Echogram Length (m)	Average Superpixel Area (m^2^)	Number of Superpixels
183	106	125
290	121	300
422	146	700
544	130	900
646	124	900

Similar analyses could be completed is other sedimentary environments. A linear regression could be used to approximate the number of super pixels required to capture the variations. For example, a linear regression of the data presented in Table 8 reveals the optimal number of superpixels is about 1.46 times the echogram length in meters.

### Recommendations for sedimentary change detection

Detecting change of surficial riverbed sediment in a mixed sand-gravel-rock alluvial channel requires the echograms be collected under similar conditions. First, the imagery between collected at two discrete points in time needs to be high quality, in order for delineation of heterogeneous riverbed into homogeneous regions of similar sized sediment. High suspended sediment concentrations impedes the transmission of sound and results in degraded imagery quality. Any echogram used for change detection needs be be collected with similar system settings (i.e. range and frequency) and approximately at the same location. Reach scale, cell-by-cell change detection is not practically achievable with recreational-grade side scan sonar echograms because positional and heading errors of the transducer translate to inexact positioning of georectified echograms [22]. Therefore, all changes in bed cover can be interpreted as redistributions of areal proportion of each sediment type over relatively broad scales and changes can only be quantified at the site/reach summary scale (i.e. reach-scale and reach-resolution). Interpreting changes in bed cover interpreted using GLCM based texture metrics requires physical context. For example, changes from low to high values within the texture features GLCM variance or Entropy can be interpreted as the bed changing from a sand-dominant to a boulder-dominant bed cover. Conversely, changes from low to high values within the texture feature Homogeneity can be interpreted as the riverbed changing from a boulder-dominant bed cover to a sand-dominant bed cover. Therefore, immobile boulders are either inundated with sand or exposed as sand is transported downstream. Reliably detecting changes in the areal fractions of gravel-dominant bed covers using GLCM texture properties is only possible if changes are sufficiently large (i.e. >> 9 m^2^) because of the relatively high uncertainty.

Suggested further work may include research into optimal geostatistical interpolation and extrapolation of substrate classes, from portions of the scans where texture segmentation is viable, to those regions of poor quality where texture segmentation is not possible. The present technique would also be amenable to site-specific calibration from independent field observations of the bed, such as from sparse video or physical samples. Finally, integrating data from repeat scans made at different sonar frequencies (for example, at 455 and 800 kHz) may enhance the ability of texturally based models such as those described here to discriminate amount substrates and bottom types.

## Summary

The textural signatures of riverbed sediment were examined using georectified echograms collected using a recreational-grade system. We identified three GLCM properties, namely Homogeneity, Entropy and GLCM variance, as metrics that can objectively quantify the textures associated with different sediment types. Broad-scale sediment classification was carried out on a regular 3×3 meter grid using two approaches: linear least-squares and GMM. Each classification approach has it own merits, but overall the GMM outperformed the least-squares approach based on its ability to estimate reach-scale proportions of different sediment types. Of the two GMM models tested presented in this paper, sand and boulder classifications could be carried out with higher accuracy than for gravel. Modeling gravel as two Gaussian density functions significantly increased the ability to correctly classify both gravel and boulders. The GMM modeling approach shows promise for application to similar sedimentary environments where there is textural variations within discrete sediment categories caused by grain size and morphological variations. Being inherently probabilistic, the GMM approach provides a measure of uncertainty for each substrate classification. The GLCM-GMM approach produces similar spatial distributions of sediment types and reach scale proportions of each sediment type compared to sediment maps compiled using multibeam backscatter. This work provides an objective methodology to develop automated and robust sediment classification algorithms using a straightforward calibration procedure by delineating echograms into perceptually meaningful regions based on their tonal and textural properties.

The demonstrated relationships between statistical descriptors of bed texture and riverbed sediment grain size present an objective means to interpret side scan sonar echograms collected from a recreational-grade system. The methods outlined in this paper, encoded in open-source and freely available software (https://github.com/danhamill/ss_texture_analysis), contribute to the ongoing democratization of recreational-grade side scan sonar technology by combining textural analysis methods with mapping methods and automated texture segmentation algorithms. In concert, these methods provide a low-cost framework for coordinated research efforts among aquatic ecologists.
